# Diabetes medication recommendation system using patient similarity analytics

**DOI:** 10.1038/s41598-022-24494-x

**Published:** 2022-12-03

**Authors:** Wei Ying Tan, Qiao Gao, Ronald Wihal Oei, Wynne Hsu, Mong Li Lee, Ngiap Chuan Tan

**Affiliations:** 1 Institute of Data Science, National University of Singapore, 3 Research Link, #04-06, Singapore, 117602 Singapore; 2 Saw Swee Hock School of Public Health, National University of Singapore, Singapore, Singapore; 3 School of Computing, National University of Singapore, Singapore, Singapore; 4 SingHealth Polyclinics, SingHealth, Singapore, Singapore; 5 Family Medicine Academic Clinical Programme, SingHealth-Duke NUS Academic Medical Centre, Singapore, Singapore

**Keywords:** Type 2 diabetes, Medical research

## Abstract

Type-2 diabetes mellitus (T2DM) is a medical condition in which oral medications avail to patients to curb their hyperglycaemia after failed dietary therapy. However, individual responses to the prescribed pharmacotherapy may differ due to their clinical profiles, comorbidities, lifestyles and medical adherence. One approach is to identify similar patients within the same community to predict their likely response to the prescribed diabetes medications. This study aims to present an evidence-based diabetes medication recommendation system (DMRS) underpinned by patient similarity analytics. The DMRS was developed using 10-year electronic health records of 54,933 adult patients with T2DM from six primary care clinics in Singapore. Multiple clinical variables including patient demographics, comorbidities, laboratory test results, existing medications, and trajectory patterns of haemoglobin A_1c_ (HbA_1c_) were used to identify similar patients. The DMRS was evaluated on four groups of patients with comorbidities such as hyperlipidaemia (HLD) and hypertension (HTN). Recommendations were assessed using hit ratio which represents the percentage of patients with at least one recommended sets of medication matches exactly the diabetes prescriptions in both the type and dosage. Recall, precision, and mean reciprocal ranking of the recommendation against the diabetes prescriptions in the EHR records were also computed. Evaluation against the EHR prescriptions revealed that the DMRS recommendations can achieve hit ratio of 81% for diabetes patients with no comorbidity, 84% for those with HLD, 78% for those with HTN, and 75% for those with both HLD and HTN. By considering patients’ clinical profiles and their trajectory patterns of HbA_1c_, the DMRS can provide an individualized recommendation that resembles the actual prescribed medication and dosage. Such a system is useful as a shared decision-making tool to assist clinicians in selecting the appropriate medications for patients with T2DM.

## Introduction

Type-2 diabetes mellitus (T2DM) is a complex, chronic illness with increased risk of premature death and disability^1^. Patients with T2DM differs in terms of their clinical profiles, comorbidities, lifestyles and medical adherence^2–4^. Pharmacotherapy for managing T2DM may involve a combination of medications to achieve optimal glycaemic control. Over the years, increasing prevalence of patients with diabetes worldwide have encouraged the development of new pharmaceutical drugs and expanded the therapeutic treatment options^5,6^. Formulating an individualized treatment plan is increasingly challenging due to heterogeneity in patients’ clinical profiles along with a growing list of new medications to prescribe. Numerous clinical practice guidelines (CPGs)^3,7,8^ exist to guide clinicians in selecting the most appropriate diabetes treatment options. However, CPGs have broad definitions of patient’s groups and a standardized one-size-fits-all treatment approach^9–11^. In addition, most CPGs provide treatment recommendations tailored to a single condition^12^ while patients with T2DM often have several comorbidities i.e. hyperlipidaemia (HLD) and hypertension (HTN)^13,14^.

To address these limitations, several artificial intelligence (AI) medication recommendation systems^15–17^ have been proposed. While these systems demonstrated the potential to make clinically relevant recommendations, their real-world deployments in clinical practice are rare due to complex logic and the lack of explanation to support recommendations^18^. Previous studies^19–22^ have shown how patient similarity analytics can enhance the interpretability of recommendation systems and assist with clinical decision making. In this study, we aim to develop an evidence-based diabetes medication recommendation system (DMRS) underpinned by patient similarity analytics for patients with T2DM. The DMRS takes into consideration the age of a patient, his/her clinical profile, comorbidities, existing medications, and trajectory of their haemoglobin A_1c_ (HbA_1c_) results.

## Methods

### Study cohort

Data used in this study was obtained from the EHR of six primary care clinics in Singapore. The study was approved by SingHealth Centralized Institutional Review Board (Reference Number: 2019/2604) prior to conduct of the study. Requirement of written consent was waived by the SingHealth Centralized Institutional Review Board as it was deemed impracticable while privacy risks were mitigated through the use of de-identified data. All methods were performed in accordance with relevant guidelines and regulations. The study cohort comprises multi-ethnic Asians adult patients, age 21 years or older having diabetes diagnosis under International Classification of Diseases (ICD), 9th or 10th Revision codes (250.90, 250.40, 250.80, E11.9, E11.21, E11.22, E14.31, E14.73 and E11.40) or have at least one diabetes medication in the 10-year period. These patients may also have hypertension (HTN) with ICD codes 401.1, 796.2, I10, or if they were on one or more anti-hypertensive medications. They may also have hyperlipidaemia (HLD) with ICD codes 272.0, E78.5, or if they were on one or more lipid-lowering medications. Patients’ demographic, disease history, laboratory test results and medications prescriptions were extracted over a 10-year period from 1 January 2010 to 31 December 2019. In total, the study cohort consisted of 54,933 patients.

### Patient variables

Variables used for patient similarity learning included age, gender, blood pressure, cholesterol, triglycerides, HbA_1c_, disease duration of diabetes (DM), HLD and HTN, medications for DM, HLD and HTN. Medications were grouped by their classes and medication counts were derived by counting the number of medications in each class. In total, there were 18 DM medication types and six DM medication classes, nine HLD medication types and four HLD medication classes and 22 HTN medication types and eight HTN medication classes. All medication dosages except Insulin were expressed in prescribed daily dose (PDD) defined using three intensity level: low (L), moderate (M) and high (H) with reference to the maximum daily dosage (for example, L: ≤ 1/3 maximum daily dosage, M: ≤ 2/3 maximum daily dosage and H: > 2/3 maximum daily dosage). The list of medication types, classes and their dosage intensity can be found in Supplementary Table S1. To avoid complexity and the risk of overtreatment of insulin with oral DM medications^23^, insulin medications were regarded as binary variable (i.e. 1 if patient was prescribed an Insulin medication, 0 otherwise).

### Recommendation algorithm

Given a target patient, the proposed DMRS uses patient’s latest clinical profiles and HbA_1c_ trajectories to retrieve a set of similar patients and use their prescribed medications for the recommendation.

For the clinical profile similarity, we used the data-driven and domain knowledge (D3K) similarity measure proposed by Oei et al.^24^ to obtain an initial similarity score. The patient variables consisted of age, gender, systolic and diastolic blood pressure, cholesterol High-Density Lipoprotein (HDL), Low-Density Lipoprotein (LDL), triglyceride, HbA_1c_, HLD and HTN comorbidities, duration of disease(s) in years and count of medication types and classes. These variables were weighted by learning a generalized Mahalanobis measure that maximized the distance between patient pair, ($P_{i}$, $P_{k}$) deemed to be clinically dissimilar while minimizing the distance between patients ($P_{i}$, $P_{j}$) deemed as clinically similar. The personalized score of patient P for variable v, denoted as score (P, v), was determined based on the P’s value for v. Given two patients P1, P2, each with D variables, their D3K similarity score was given by

$$D3K\_sim\left( {P1,P2} \right) = \frac{{2\mathop \sum \nolimits_{v = 1}^{D} \min \left( {score{ }\,\left( {P1,{ }v} \right),{ }score{ }\left( {P2,{ }v,} \right)} \right)}}{{(\mathop \sum \nolimits_{v = 1}^{D} score\,\left( {P1,v} \right) + \mathop \sum \nolimits_{v = 1}^{D} score\left( {P2,v} \right))}}$$

For the management of chronic diseases such as diabetes, we observed that besides assessing whether the patient’s HbA_1c_ value falls within the normal range, clinicians often up or down-titrate medication dosage with regards to the trend in HbA_1c_ test results over the years. We modelled how clinicians typically analysed patient’s lab results by mapping patient’s HbA_1c_ trajectory to a sequence of symbols. The symbols represent whether the results were normal (N), abnormal (A), increasing (U) or decreasing (D). Trajectory mapping were illustrated using the two cases as follows: First, we compared the HbA_1c_ values v1 and v2 at every two consecutive months and used τ to denote a pre-determined threshold.

- Case 1. Difference between v1 and v2 ≤ τ.

If v1 lies within the normal HbA_1c_ range, we mapped this to the symbol “N”, otherwise it would be mapped to the symbol “A”.

- Case 2. Difference between v1 and v2 > τ.

If v1 < v2, we mapped this to the symbol “U” to represent an uptrend trajectory. Otherwise, we mapped to the symbol “D” to represent a downtrend trajectory.

If patients’ the HbA_1c_ value were missing for that month, linear interpolation was used to estimate the missing value. In the instance where patients’ have multiple HbA_1c_ values in a month, the average HbA_1c_ value will be considered. For example, consider a patient having normal HbA_1c_ values for three consecutive months, followed by increasing HbA_1c_ values in the next two months and remains in the abnormal range in the last two months. The patient’s HbA_1c_ trajectory will be illustrated by the sequence, ‘NNNUUAA’. Using the HbA_1c_ trajectory mapping, we counted the occurrences of each n-grams in the sequence. We used n = 6 since most patients have their HbA_1c_ tested every three to six months. There were two 6-grams in the above mapped sequence (i.e. ‘NNNUUA’ and ‘NNUUAA’) and both had a count of one.

We let d denote the total number of 6-grams in the mapped sequence of all the patients’ HbA_1c_ trajectories. A d-dimensional vector was used to represent a patient’s trajectory with each dimension corresponds to a 6-gram. The ith entry represents the count of the occurrences of the ith 6-gram. The similarity between two patients P1, P2 with trajectory vectors u and v were computed as follows:

$$traj\_sim\left( {{\text{P}}1,{\text{ P}}2} \right) = \frac{1}{{1 + \left| {\left| {u - v} \right|} \right|_{2} }}$$

The overall similarity, trajectory-D3K (T-D3K) of patients P1, P2 was determined as follows:

$$sim\left( {{\text{P}}1,{\text{P}}2} \right) = {\text{ max}}\,\left( {D3K_{{sim\left( {{\text{P}}1,{\text{P}}2} \right)}} , traj_{{sim\left( {{\text{P}}1,{\text{P}}2} \right)}} } \right)$$

The proposed DMRS used the T-D3K method to generate the candidate set of medications for the target patient by retrieving prescription records of the similar patients (sorted by their overall similarity scores). For patients with the same similarity score, their medication lists were prioritized according to the degree of overlap with the target patient’s list of medications and dosages. In the instance of a tie, we ranked the medication lists based on the number of therapies (monotherapy was preferred over higher number of therapy), and dosage (lower dosage was preferred over higher dosage).

### Evaluation

The proposed DMRS was evaluated on four groups of patients with different comorbidities profile (DM only, DM with HLD, DM with HTN, and DM with HLD and HTN (DHL)). For each group, 100 test patients were randomly selected among those with suboptimal HbA1c (i.e. HbA_1c_ ≥ 8) and have at least one DM medication adjustment. For each test patient, the top K candidate medications were retrieved and ranked as described in the “Method” section.

We used the prescribed DM medication type and dose in the EHR records as our ground truth and evaluated the accuracy of our recommendations using three metrics: hit ratio, recall@K, and precision@K. Hit ratio refers to the percentage of patients where at least one of the recommended sets of medication matched the ground truth in both type and dosage.

As described in the previous section, the sets of medications to be recommended for a target patient were obtained from the prescription records of the similar patients. Each target patient can receive up to K unique sets of recommended medication.

We let S denote one set of recommended medication and G denote the ground truth medication. The percentage of matches between the recommended medication in S and that in the ground truth G were measured by recall (S,G) and precision (S,G) as follows:

$$recall\left( {S, G} \right) = \frac{{\left| {G \cap S} \right|}}{\left| G \right|}$$

$$precision\left( {S,G} \right) = \frac{{\left| {G \cap S} \right|}}{{\left| { S} \right|}}$$

For each target patient, we computed recall@K as the maximum recall (S,G) and precision@K as the maximum precision (S,G) *a*mong the K sets of recommended medication. In addition, to evaluate the quality of the recommendations, we used Mean Reciprocal Rank (MRR)^25^. MRR takes into account the position of the matched recommendation in the list. A high MRR indicates that the matched recommendation was positioned near the top of the recommendation list.

We compared our T-D3K approach of medication recommender systems with the following methods:

- Euclidean distance on normalized data.
- D3K. This approach retrieves similar patients and their prescribed medications using multiple clinical measurements and medication counts at a single time point.
- Trajectory. This approach retrieves similar patients and their prescribed medications based on HbA1c (%) trajectory over multiple time points.

### Ethics approval

Ethics approval was obtained from SingHealth Centralized Institution Review Board (CIRB) in 2019 (SingHealth CIRB Reference: 2019/2604). Patient consent was not obtained as the analysis was conducted on de-identified data.

## Results

### Patients’ characteristics

Patients’ characteristics based on their latest EHR across the 10-year observation period were shown in Table 1. The study cohort consisted of 54,933 diabetic patients with mean disease duration of 5.3 ± 2.1 years. 7.6% of the patients had a comorbidity of hyperlipidaemia (DM with HLD), 2.7% had hypertension (DM with HTN) and 88.7% had both hyperlipidaemia and hypertension (DHL). The mean age of patients were 67.4 ± 11.4 years with 49.2% being male. The cohort was predominantly Chinese (69.4%) followed by Malay (16.0%), followed by Indian (9.9%), which is similar to the ethnic distribution in the local Asian population. The average systolic and diastolic blood pressure (mmHg) were 133.0 ± 6.7 and 69.0 ± 9.4 respectively. Average cholesterol LDL mmol/L and HbA_1c_% were 2.2 ± 0.9 and 7.3 ± 1.3 respectively. Majority of the patients were on mono (42.1%) and dual diabetic medications (30.2%). More than 90% of the patients have at least one or more DM medication adjustment during their 10 years of visit records.

**Table 1:** Characteristics of patients in the study cohort.

Variables	Overall n = 54,933
Age, years	67.4 ± 11.4
Male (%)	27,018 (49.2)
Race (%)	
Chinese	38,150 (69.4)
Indian	5,429 (9.9)
Malay	8,789 (16.0)
Others	2,565 (4.7)
**Comorbidities (%)**	
Diabetes (DM only)	567 (1.0)
Diabetes and hyperlipidaemia (DM with HLD)	4,162 (7.6)
Diabetes and hypertension (DM with HTN)	1,505 (2.7)
Diabetes, hyperlipidaemia and hypertension (DHL)	48,699 (88.7)
**Disease duration, years**	
Diabetes (DM)	5.3 ± 2.1
Hyperlipidaemia (HLD)	5.5 ± 1.9
Hypertension (HTN)	5.5 ± 1.9
Blood pressure systolic (mmHg)	133.0 ± 16.7
Blood pressure diastolic (mmHg)	69.0 ± 9.4
Cholesterol high-density lipoprotein (HDL) (mmol/L)	1.3 ± 0.4
Cholesterol low-density lipoprotein (LDL) (mmol/L)	2.2 ± 0.9
Triglycerides (mmol/L)	1.6 ± 1.1
Haemoglobin A1c level (HbA_1c_) (%)	7.3 ± 1.3
**Medications**	
Diabetes (DM) medication count^1^	
One medication	23,127 (42.1)
Two medications	16,587 (30.2)
Three or more medications	15,219 (27.7)
Hyperlipidaemia (HLD) medication count^1^	
No medication	5,838 (10.6)
One medication	43,910 (79.9)
Two medications	5,077 (9.2)
Three or more medications	108 (0.2)
Hypertension (HTN) medication count^1^	
No medication	8,559 (15.6)
One medication	16,819 (30.6)
Two medications	17,498 (31.9)
Three or more medications	12,057 (21.9)

^1^Medication count denotes count of patients on zero, one, two or three or more medications. Continuous variables were expressed as a mean value ± SD, while categorical variables expressed as the number of patients, n (%).

### Medication recommendations

Results for the four groups of patients at K = 10 was shown in Table 2. Across the four groups of patients, we found that T-D3K consistently recorded the highest hit ratio and MRR. Averaged hit ratio, recall@10 and MRR for T-D3K were 79.50%, 0.94 and 0.52 respectively. D3K recorded the best averaged precision@10 at 0.98, approximately 2.6% higher than T-D3K (0.95).

**Table 2:** Comparison of the Hit ratio, Recall@10, Precision@10 and MRR of the various methods for the four groups of patients.

Patient group	Method	Hit ratio	Recall@10	Precision@10	MRR
DM^1^ only	Euclidean	67	0.854	0.877	0.431
	D3K	76	0.923	**0.975**	0.474
	Trajectory	73	0.938	0.944	0.383
	T-D3K	**81**	**0.946**	0.954	**0.551**
DM with HLD^2^	Euclidean	63	0.849	0.901	0.378
	D3K	82	**0.945**	**0.998**	0.455
	Trajectory	70	0.905	0.94	0.367
	T-D3K	**84**	0.935	0.954	**0.526**
DM with HTN^3^	Euclidean	65	0.876	0.888	0.316
	D3K	76	0.907	**0.990**	0.397
	Trajectory	75	0.925	0.947	0.408
	T-D3K	**78**	**0.927**	0.945	**0.534**
DHL^4^	Euclidean	54	0.823	0.862	0.301
	D3K	61	0.879	0.944	0.303
	Trajectory	67	0.902	0.953	0.397
	T-D3K	**75**	**0.940**	**0.956**	**0.452**
Average of four patient groups	Euclidean	62.25	0.851	0.882	0.357
	D3K	73.75	0.914	**0.977**	0.407
	Trajectory	71.25	0.918	0.946	0.389
	T-D3K	**79.5**	**0.937**	0.952	**0.516**

^1^DM denotes patients with T2DM only, DM with HLD^2^ denotes T2DM patients with hyperlipidaemia (HLD), DM with HTN^3^ denotes T2DM patients with hypertension (HTN), and DHL^4^ denotes T2DM patients with hyperlipidaemia and hypertension (DHL). ^5^Hit ratio % is the proportion patients with recommendations that have an exact match (both the type and dosage) with prescribed medications. ^6^Recall measures the number of common medication type and dose between recommended set (S) and ground truth set (G) over ground truth set (G). ^7^Precision measures the number of common medication type and dose between recommended set (S) and ground truth (G) over recommended set (S). [Bold] figures represent the best performance among the four similarity approach for each comorbidities profile.

Comparing the performance of T-D3K across the four patient groups, hit ratio was the highest for patient group DM with HLD (84%) followed by patient group with DM only (81%), DM with HTN (78%) and DHL (75%). This suggest that out of the 100-sample patient, T-D3K provided recommendations that matched the actual prescriptions for at least 75 patients. Performance of T-D3K in recall@10 and precision@10 were largely similar across the four patient groups. Recall@10 and precision@10 for T-D3K ranged between 0.93–0.95 and 0.95–0.96 respectively. MRR for T-D3K was above 0.5 for all patient groups except for patient with DHL. A lower MRR indicates that T-D3K tend to make the correct recommendations at mid to bottom position of the recommendations list for the DHL patients group.

**Figure 1:**
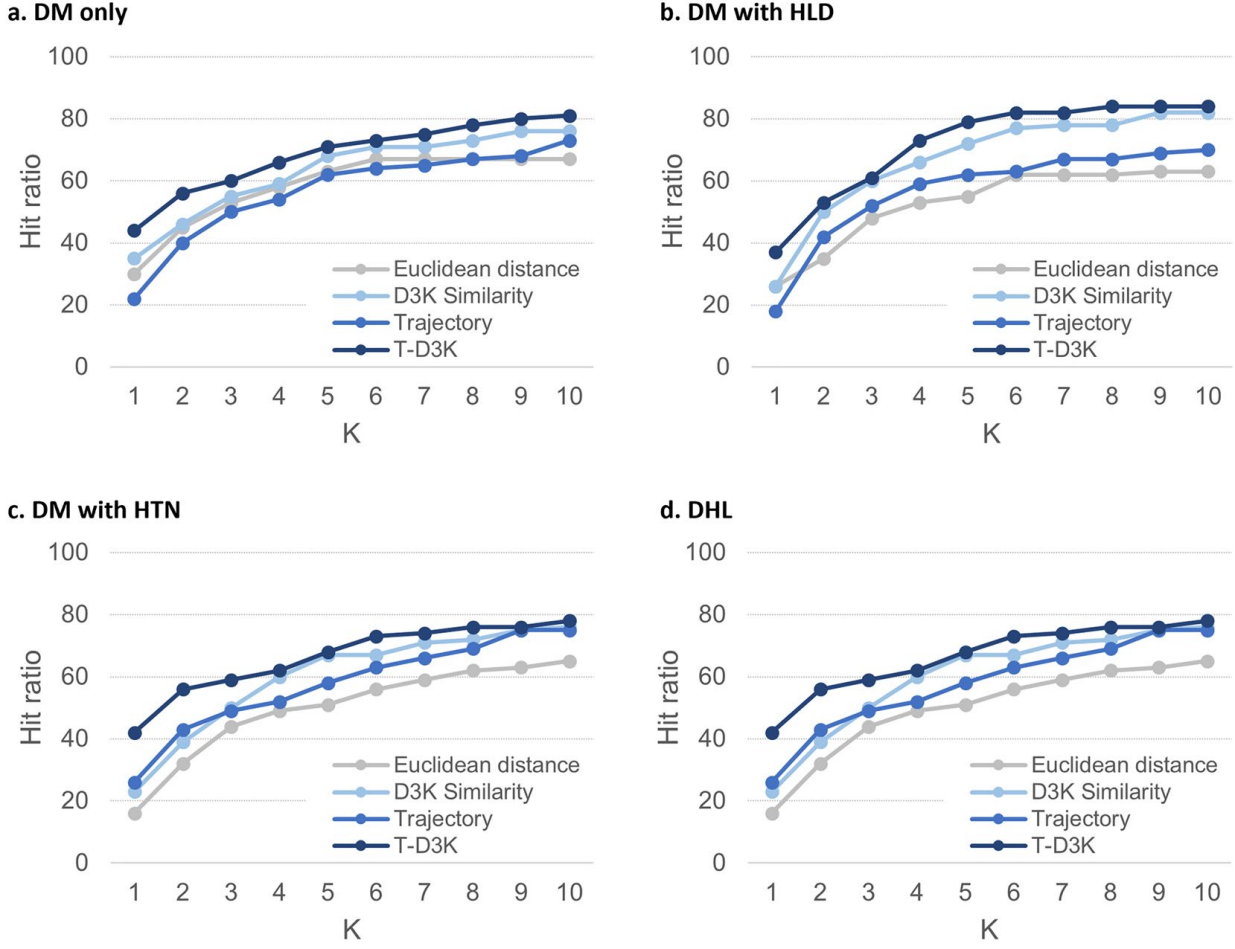
Hit ratio for the four groups of patients.

Figure 1 shows the hit ratio as we vary K for the four groups of patients. Overall, T-D3K approach achieved the highest hit ratio. At K = 10, the hit ratios of T-D3K were 81%, 84%, 78% and 75% for patient groups DM only, DM with HLD, DM with HTN, and DHL respectively.

**Figure 2:**
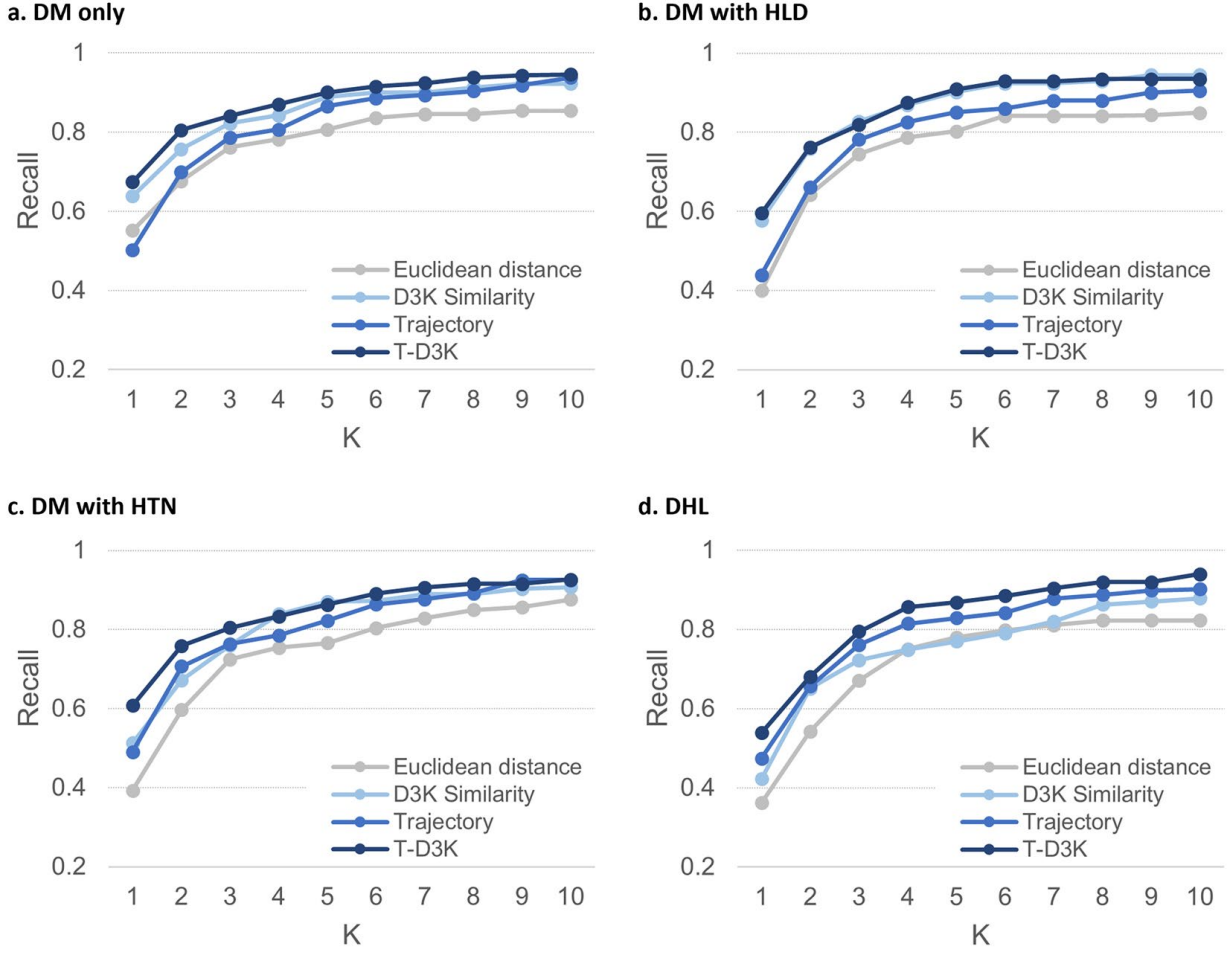
Recall for four groups of patients.

Figure 2 shows the recall@K as we vary K for all four patient groups. T-D3K had the best performance for all four patient groups except patient group with DM with HLD. D3K recorded the highest recall for patient group DM with HLD at 0.95. Specifically, recall@10 for T-D3K were 0.95, 0.94, 0.93, 0.94 for patient groups DM only, DM with HLD, DM with HTN, and DHL respectively.

**Figure 3:**
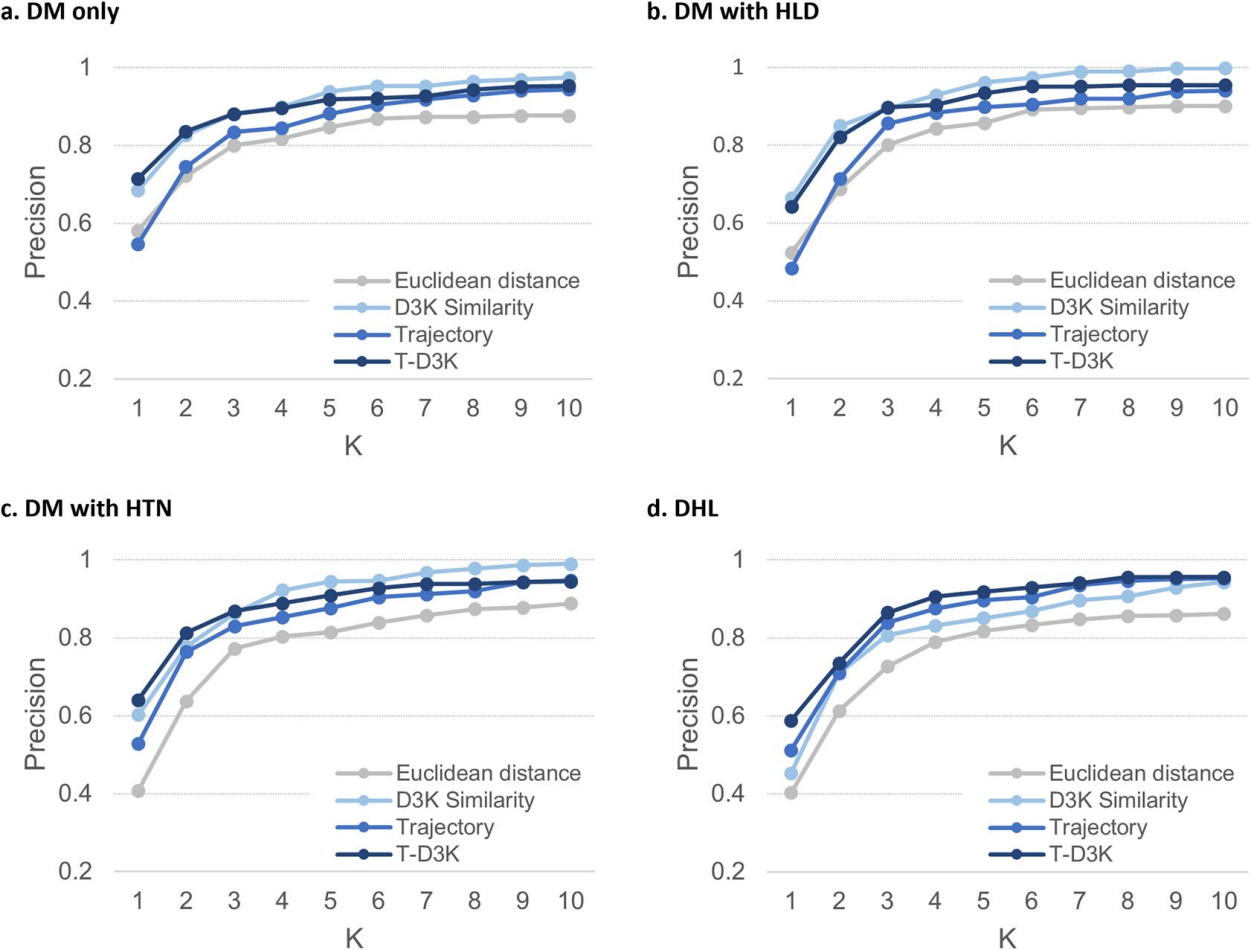
Precision for four groups of patients.

Figure 3 shows the precision@K for the four patient groups. T-D3K delivered the best performance for the DHL patient group, while D3K recorded the best precision for patient groups DM only, DM with HLD, DM with HTN. When K = 10, T-D3K had a precision of 0.95 for patient groups DM only, DM with HLD and DM with HTN, and 0.96 for DHL patient group.

**Figure 4:**
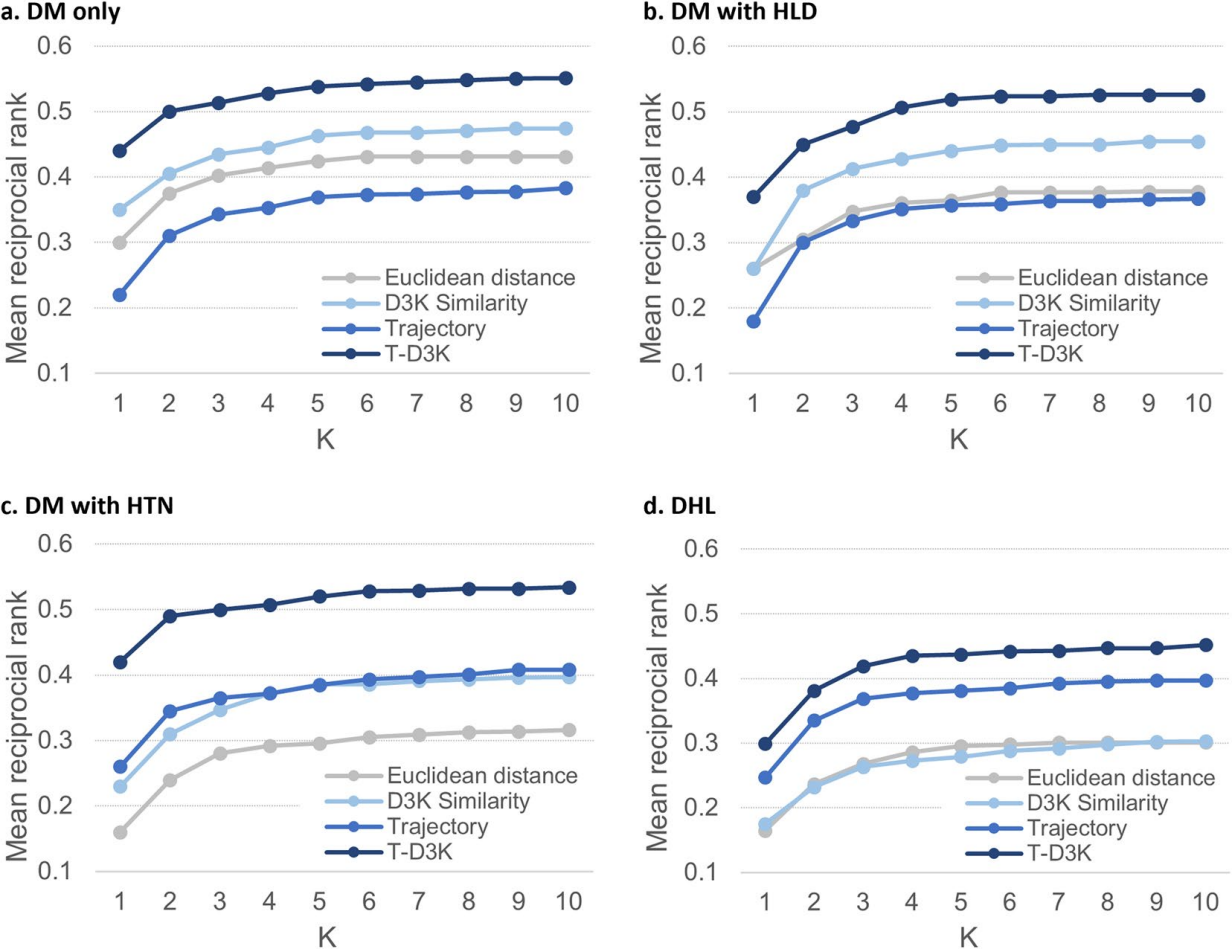
Mean reciprocal rank (MRR) for four groups of patients.

Figure 4 shows the MRR results with T-D3K having the highest MRR for all the groups. When K = 10, MRR for T-D3K were 0.55, 0.53, 0.53 and 0.45 for patient groups DM only, DM with HLD, DM with HTN, and DHL respectively.

## Discussion

In this work, we proposed a DMRS using patients’ clinical profiles and their HbA_1c_ test results trajectory. Our system used similar patient analytics to personalize the recommendations as we found that patients with clinically similar profile and diabetes disease trajectories were likely to share similar prescribed medication types and dosages. The set of similar patients retrieves also provided some form of explanation for the recommendation, thus enhancing system transparency, interpretability and trust for use in clinical practice.

Previous works^26,27^ on medicine recommendation system developed using patient similarity approach were mostly derived from a single time point. However, studies^28–30^ have shown that HbA_1c_ trajectories (i.e. the delayed or metabolic memory) of patients with T2DM have important effects on diabetes outcomes. Non-stable HbA_1c_ trajectories are associated with greater risk of microvascular events and mortality^28^. This study extends existing works by considering patients’ HbA_1c_ trajectories in addition to clinical profile when identifying similar patients. In contrast to studies^20,21^ that provide recommendations for medications type only, our study provides both medication type and dosage recommendations. Integrating dosage recommendation increases the complexity of medication recommendation system as titration of dosages need to take into consideration the age of a patient, their clinical profile, and/or other interacting medications^31^.

Among the four patient groups, patients in DHL group recorded the lowest hit ratio and MRR. This result was consistent across the different methods. One possible explanation is that individualizing medication recommendations for patients with multiple, concurrent chronic conditions can be complex and challenging where the treatment for one condition may interfere with the treatment of other conditions^32^.

This study has several limitations. First, our study utilized EHR data from a community healthcare system. Hence, diabetes prescriptions were influenced by availability of government subsidies. Only medications classified under the standard drug list (SDL) were eligible for government subsidy^33^. As a result, biguanides and sulfonylureas prescriptions were overrepresented in EHR since they fall under SDL and were used as the first-line treatment for patients with suboptimal HbA_1c_^34^. Second, the DMRS was developed using the EHR of six primary care clinics over a 10-year period. This may result in the recommendation of dated medications, e.g., use of Tolbutamide as Sulfonylurea agent. Further, the recommended medications tend not to include new drugs classes such as Dipeptidyl peptidase-4 (DPP-4) inhibitors and Sodium-glucose cotransporter-2 (SGLT-2) inhibitors which may reduce pill burden and improve patient adherence^3^. Third, dosage recommendations in this work were limited to three intensity level (low, medium and high) so as to reduce the computational overhead. Lastly, insulin was regarded as a binary medication variable. Hence, the DMRS is best applied to clinical care of patients before secondary drug failure (i.e. failure of oral medications to maintain optimal glycaemic control).

### Clinical utility

The DMRS is currently integrated as part of a module along with other diabetes risk stratification and prognostication modules in an easy-to-use web interface known as PERsonalized DIabetes Counselling Tool using Artificial Intelligence (PERDICT.AI)^35^. PERDICT.AI aims to support clinicians in diabetes consultations and to improve medication optimization. To use the DMRS, clinicians can either manually input or enter only the patient’s identity number to automatically populate the patient’s clinical profile (such as age, gender, blood pressure, cholesterol, HbA_1c_ and existing medications). Using patient’s latest clinical profiles and HbA_1c_ trajectories, the DMRS will run at the backend of the PERDICT.AI interface, to retrieve a set of similar patients and their prescribed medications for diabetes medicine recommendation. The clinical application of PERDICT.AI will be tested at several primary care clinics in Singapore. The pilot study of the PERDICT.AI will induct patients with suboptimal glycaemic control via patient similarity approaches, which allows their understanding of their individual risk profiles, followed by recommendations of suitable pharmacotherapy using the DMRS. The mix-method study and the outcomes will be shared when the study is completed.

## Conclusion

The proposed DMRS is able to provide an individualized recommendation that is close to actual prescribed medication and dosage by taking into consideration patient’s clinical profile and glycaemic control trajectories. Such a system is useful as a shared decision-making tool to assist clinicians in selecting the appropriate medications for patients with T2DM.

## Supplementary Information

Supplementary Information.

## Data Availability

The datasets analysed in the current study are not publicly available as they contain information that are sensitive to the institution. They may be made available from the corresponding author on reasonable request.

## Abbreviations

CPG: Clinical practice guidelines
D3K: Data-driven and domain knowledge
DPP-4 inhibitor: Dipeptidyl peptidase-4 inhibitor
DM: Diabetes mellitus
DHL: Diabetes, hyperlipidaemia and hypertension
EHR: Electronic health records
HbA_1c_: Haemoglobin A_1c_
HDL: Cholesterol high-density lipoprotein
HLD: Hyperlipidaemia
HTN: Hypertension
ICD: International classification of diseases
LDL: Cholesterol high-density lipoprotein
PDD: Prescribed daily dose
SGLT-2 inhibitor: Sodium-glucose cotransporter-2 inhibitor
SDL: Standard drug list
T2DM: Type-2 diabetes mellitus
