# Supplementary material for: Diabetes medication recommendation system using patient similarity analytics
Source: Sci Rep. 2022 Dec 3;12:20910. doi: 10.1038/s41598-022-24494-x (PMC9719534; doi:10.1038/s41598-022-24494-x)
Supplement: Supplementary file 1 — Supplementary Information. [file 41598_2022_24494_MOESM1_ESM.docx]

**Diabetes medication recommendation system using patient similarity analytics**

**Supporting information**

**S1 Table. List of diabetic drugs, medication class and their dose intensity.**

| Medication class | Diabetic medications | Low  intensity | Medium intensity | High  intensity |
| --- | --- | --- | --- | --- |
| Alpha-glucosidase inhibitors | Acarbose | 100 | 200 | 300 |
| Biguanides | Metformin | 1,000 | 2,000 | 3,000 |
| Sulfonylureas | Gliclazide | 80 | 160 | 320 |
|  | Glipizide | 10 | 20 | 30 |
|  | Tolbutamide | 1,000 | 2,000 | 3,000 |
| DPP-4 inhibitors | Linagliptin | - | 5 | - |
|  | Sitagliptin | 25 | 50 | 100 |
|  | Vildagliptin | 25 | 75 | 100 |
| SGLT-2 inhibitors | Dapagliflozin | 5 | 10 | 25 |
|  | Empagliflozin | 10 | 12.5 | 25 |
| Insulin^1^ | Binary variable | | | |

^1^ Insulin consist of Actrapid, Apidra, Insulatard, Lantus, Levemir, Mixtard, Novomix and Novorapid. Insulin is regarded as a binary medication.
